# ClinicalTrials.gov registration can supplement information in abstracts for systematic reviews: a comparison study

**DOI:** 10.1186/1471-2288-13-79

**Published:** 2013-06-18

**Authors:** Roberta W Scherer, Lynn Huynh, Ann-Margret Ervin, Jakeisha Taylor, Kay Dickersin

**Affiliations:** 1 Center for Clinical Trials, Department of Epidemiology, Johns Hopkins Bloomberg School of Public Health, Baltimore, MD 21205, USA; 2 Baptist Desoto Hospital, Southaven, MS 38671, USA

## Abstract

**Background:**

The inclusion of randomized controlled trials (RCTs) reported in conference abstracts in systematic reviews is controversial, partly because study design information and risk of bias is often not fully reported in the abstract. The Association for Research in Vision and Ophthalmology (ARVO) requires trial registration of abstracts submitted for their annual conference as of 2007. Our goal was to assess the feasibility of obtaining study design information critical to systematic reviews, but not typically included in conference abstracts, from the trial registration record.

**Methods:**

We reviewed all conference abstracts presented at the ARVO meetings from 2007 through 2009, and identified 496 RCTs; 154 had a single matching registration record in ClinicalTrials.gov. Two individuals independently extracted information from the abstract and the ClinicalTrials.gov record, including study design, sample size, inclusion criteria, masking, interventions, outcomes, funder, and investigator name and contact information. Discrepancies were resolved by consensus. We assessed the frequencies of reporting variables appearing in the abstract and the trial register and assessed agreement of information reported in both sources.

**Results:**

We found a substantial amount of study design information in the ClinicalTrials.gov record that was unavailable in the corresponding conference abstract, including eligibility criteria associated with gender (83%; 128/154); masking or blinding of study participants (53%, 82/154), persons administering treatment (30%, 46/154), and persons measuring the outcomes (40%, 61/154)); and number of study centers (58%; 90/154). Only 34% (52/154) of abstracts explicitly described a primary outcome, but a primary outcome was included in the “Primary Outcome” field in the ClinicalTrials.gov record for 82% (126/154) of studies. One or more study interventions were reported in each abstract, but agreed exactly with those reported in ClinicalTrials.gov only slightly more than half the time (88/154, 56%). We found no contact information for study investigators in the abstract, but this information was available in less than one quarter of ClinicalTrial.gov records (17%; 26/154).

**Conclusion:**

RCT design information not reported in conference abstracts is often available in the corresponding ClinicalTrials.gov registration record. Sometimes there is conflicting information reported in the two sources and further contact with the trial investigators may still be required.

## Background

Identification of relevant randomized controlled trials (RCTs) is an integral part in the conduct of systematic reviews of intervention efficacy and effectiveness. RCTs are usually identified through searching electronic databases (e.g., PubMed, EMBASE) and handsearching (manually screening biomedical journals, conference proceedings and other publications). Recently, the Institute of Medicine (IOM) published guidelines stating that results from conference abstracts should be included in systematic reviews because conference abstracts provide an important source of unpublished trials [1]. This is because only about 60% of controlled clinical trials presented as conference abstracts are subsequently published as journal articles, and trials with negative or null results are published less frequently than those with positive findings [2]. The end result is that inclusion of the grey literature in systematic reviews and meta-analyses leads to smaller treatment effect sizes overall [3]. This presents a problem for systematic reviewers, however, as reporting of study design in abstracts is poor [4] and results reported are often preliminary. Furthermore, there are concerns that abstracts rarely undergo peer review. Although adoption of CONSORT reporting guidelines for conference abstracts may lead to some improvement over time, controversy remains about whether to include conference abstracts in systematic reviews [5-7].

One approach to learning more about critical design elements of trials reported only in conference abstracts is to seek information from other sources. For example, a trials register such as ClinicalTrials.gov includes key protocol items relevant to determining eligibility and performing critical appraisal of a study.

We hypothesized that information included in a trials register record could be used to supplement the sparse information on study design presented in a conference abstract. To test the hypothesis, we obtained reports of RCTs presented at the annual meeting of the Association for Research in Vision and Ophthalmology (ARVO) from 2007 to 2009; this is an international meeting of more than 10,000 attendees. The ARVO organizers require that any abstract submitted for presentation at the annual meeting that describes a concurrently controlled trial must be registered in an electronically searchable, publicly available trials register [8]. The online conference abstract submission form includes a box in which abstract authors reporting controlled clinical trials are asked to supply the name of the trials register and the trial registration number where the trial was registered. Accompanying instructions include a definition of a clinical trial and a hyperlink to a frequently asked questions page that describes trials registers, including a drop-down menu of acceptable registers. A previous study showed that when authors complied with this requirement, about 90% reported registering trials at ClinicalTrials.gov [9].

## Methods

### Identification of included randomized controlled clinical trials

We reviewed all abstracts presented at the ARVO meetings from 2007 through 2009 (submitted through December 2008). Abstracts were classified as RCTs using the definition provided in the Cochrane Collaboration’s Handsearching Training Manual: “a study in which individuals (or other units) followed in the trial were definitely assigned prospectively to one of two (or more) alternative forms of health care using random allocation” [10]. One person hand searched the ARVO annual meeting abstracts online at http://www.arvo.org. A second person reviewed all studies classified as an RCT by the handsearcher and another person reviewed a sample of abstracts not classified as an RCT [9]. Discrepancies were resolved by consensus.

Two individuals independently extracted information about trial registration as reported for each abstract, including the name of the organization listed in the trial registration box and information in the box designated for the registration identification number. Abstracts listing more than one registration number or not listing any number were excluded from further analysis. Because 88% (276/312) of RCTs reporting a trials register listed ClinicalTrials.gov as the trials register [9], we chose to include only ClinicalTrials.gov records in our study. We entered the trial register number provided in the search box at http://www.clinicaltrials.gov and classified numbers as valid if a matching trial was identified in ClinicalTrials.gov, and invalid if the search yielded no results. We did not attempt to identify a ClinicalTrials.gov registration record for trials whose investigators had not included a registration number. We retrieved all ClinicalTrials.gov records for trials as posted (i.e., including amendments incorporated in the record at the time of retrieval) from May and June, 2009.

Two persons independently reviewed the abstract-ClinicalTrials.gov pairs for inclusion. We excluded abstract-ClinicalTrials.gov pairs that described secondary analyses of trial data (e.g., analyses of ancillary study data), nested case–control studies from RCT data, and methodological studies associated with the RCT, because the objective of our study was to compare descriptions of the original RCT design; we also excluded pairs where the ClinicalTrials.gov record stated that assignment to treatment was not randomized.

### Abstraction of study design characteristics

We extracted information from both the abstract and the ClinicalTrials.gov record, including type of randomized comparison (parallel, cross over, cluster), multi-center status (yes/no), number randomized, and inclusion criteria related to demographic characteristics of the study population (i.e., adults, children, included sexes, presence of a disease or condition, and/or healthy volunteers). Masking was characterized separately as yes/no for study participants, treatment administrators, and outcome assessors. The study intervention, primary outcome, secondary outcomes, and study funder(s) were extracted verbatim from the abstract and the ClinicalTrials.gov record. We classified an outcome as the “primary” outcome in the abstract only if it was explicitly stated as such, and in the ClinicalTrials.gov record only if it was included in the “Primary Outcome” field in the tabular view of the record. We classified all other outcomes reported as “non-primary” outcomes. The study funder was abstracted from the “Support” field of the abstract and either the “Sponsor” or “Collaborator” field in the ClinicalTrials.gov record. We classified funders as industry or non-industry. If the study funder was unclear, we classified it as non-industry as a conservative measure. We also collected information about study status as reported in ClinicalTrials.gov (“not yet recruiting”, “recruiting”, “active”, or “not recruiting, completed”). If more than one abstract had the same registration number, we extracted information from all relevant abstracts and reported it on a single data abstraction form. We also extracted the presence of a contact name, telephone number, and e-mail address from the ClinicalTrials.gov record. All data were abstracted independently by two abstractors (RWS, LH, KD, AE, JT) on pre-tested paper data collection forms; we extracted all information from the abstract before we extracted information from the ClinicalTrials.gov record. Discrepancies were resolved by consensus. All data from the paper forms were entered into an Access database.

### Data analyses

We assessed frequencies of reporting variables in the abstract and the trials register. We assessed the concordance between study design characteristics that were described in both the conference abstract and the trials register record by comparing reports of variables across abstract-ClinicalTrials.gov pairs. We determined whether a design characteristic that was not described in the abstract was present in the trials register. We also compared information reported in both sources and characterized the level of agreement as full, partial, or no agreement. If the abstract and the ClinicalTrials.gov record agreed exactly or nearly exactly, we classified this as full agreement. A more precise definition of an outcome in the abstract (or register) was classified as partial agreement while a completely different outcome in the abstract was classified as a new outcome*.* We further categorized partial agreement by type of disagreement and source of additional information (i.e., the abstract or the ClinicalTrials.gov record). We used SAS Version 9.2 (SAS Institute, Carey, NC) to perform all analyses.

## Results

### Registration of clinical trials

**Figure 1:**
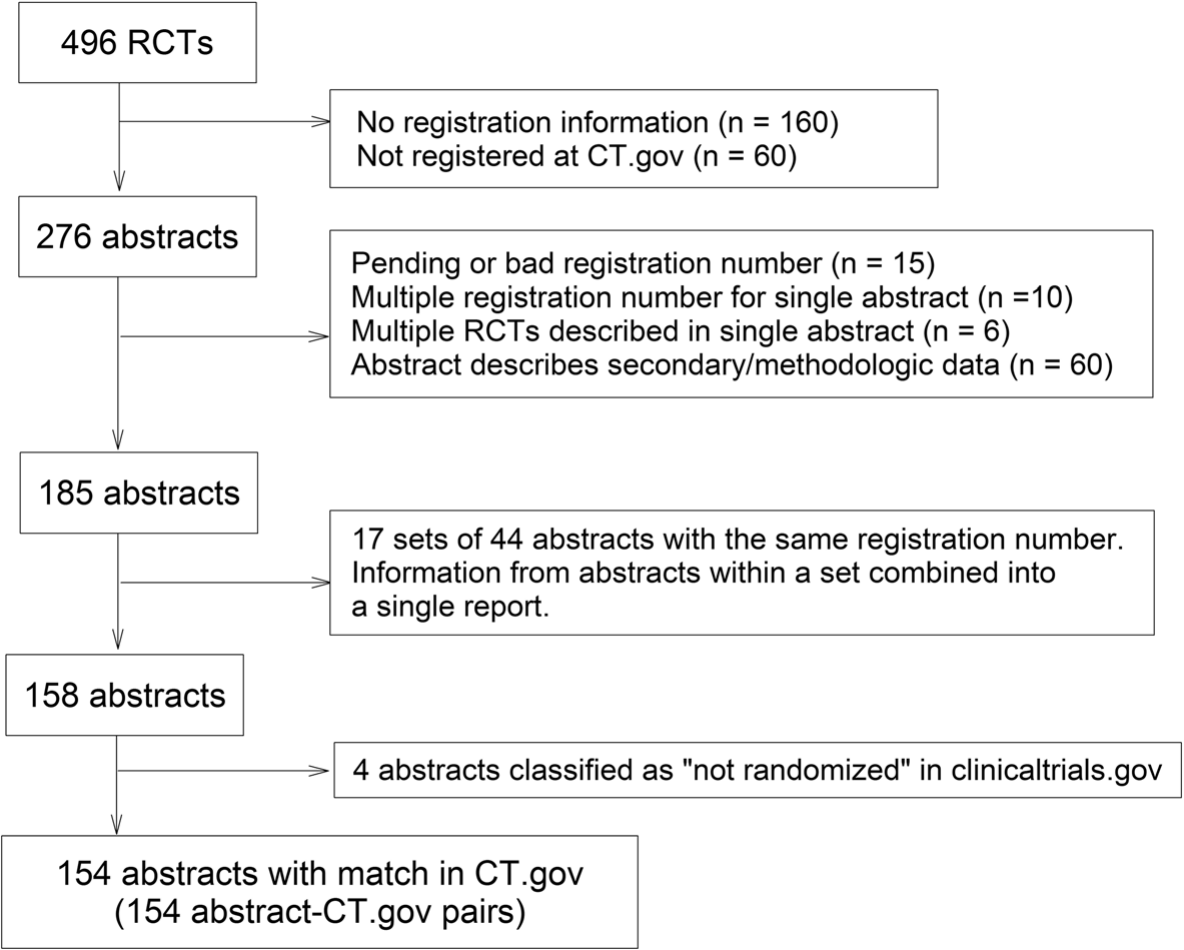
**Flow chart of conference abstract-ClinicalTrial.gov register pairs used for comparison of randomized controlled trial characteristics.** RCT = randomized controlled trial; CT.gov = ClinicalTrials.gov.

The handsearching results have previously been reported [9]. Only 2.8% (496/17,953) of all abstracts presented at ARVO from 2007 to 2009 described results of an RCT; 276 of these reported registration in ClinicalTrials.gov. Excluding abstracts not meeting our eligibility criteria resulted in 158 abstracts. We also excluded 4 abstracts in which the ClinicalTrials.gov record classified the study as not randomized. We linked each of the remaining abstracts with a single ClinicalTrials.gov registration number, resulting in 154 abstract-ClinicalTrials.gov pairs for analysis (see Figure 1).

### General study design

We observed generally good agreement on the randomized intervention comparison described in the abstract with what was reported in ClinicalTrials.gov (80.5%, 124/154) (see Table 1). Whether a trial was single or multi-center was the same in 39 pairs, different in 13, and not reported in either source for 12 pairs. For the remaining 90 pairs, no information on multi-center status was available in the abstract, but was reported in ClinicalTrials.gov.

**Table 1:** Agreement between conference abstract and ClinicalTrials.gov register on design of randomized comparison (n = 154 abstract-ClinicalTrials.gov pairs)

**Abstract**	**ClinicalTrials.gov**						
	**Parallel**	**Cross-over**	**Cluster**	Within person^*^	**Factorial**	**Other**	**Total number of pairs**
**Parallel**	**97**	5	0	1	2	7	**112**
**Cross-over**	1	**20**	0	0	1	0	**22**
**Cluster**	0	0	**1**	0	0	0	**1**
Within person*	8	1	0	**6**	0	1	**16**
**Factorial**	0	0	0	0	**0**	0	**0**
**Other**	2	1	0	0	0	**0**	**3**
**Total number of pairs**	**108**	**27**	**1**	**7**	**3**	**8**	**154**

* Trials in which the unit of randomization is not the individual, but an organ or part of the body (e.g., eyes).

### Inclusion criteria

**Figure 2:**
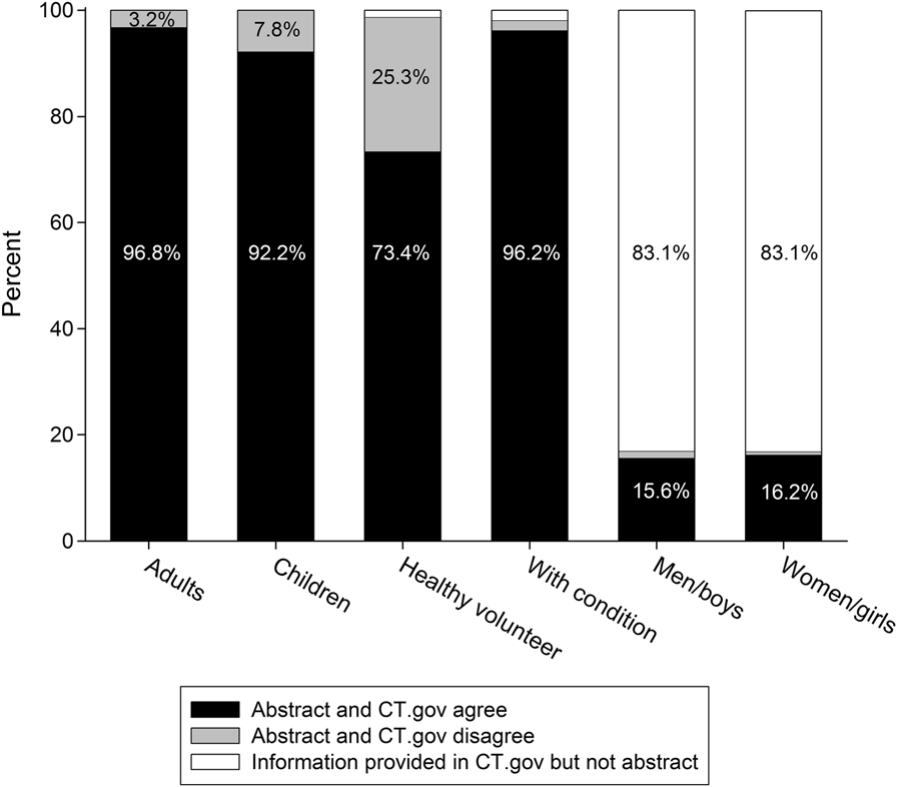
**Agreement between abstract and ClinicalTrials.gov register on eligibility criteria.** Bars show percent of abstract-ClinicalTrials.gov pairs that agree (black) or disagree (gray) on eligibility criterion, or where information on a criterion was provided in the ClinicalTrials.gov record but not the abstract (white). Eligibility criteria assessed are inclusion of adults, children, healthy volunteers, presence of a condition, men and/or boys, and women and/or girls and were categorized as present or not present. CT.gov = ClinicalTrials.gov.

There was agreement between the abstract and ClinicalTrials.gov record on inclusion criteria related to age, the presence of a disease or condition, and whether the trial participants were healthy volunteers (see Figure 2). The inclusion criterion of sex was rarely reported in the abstract, but when it was reported, the information usually agreed with what was reported in ClinicalTrials.gov. In contrast, the inclusion of men and/or women was always included in the ClinicalTrials.gov record.

### Intervention

At least one experimental and one control intervention was reported in both the abstract and the ClinicalTrials.gov record. We observed good agreement in the intervention reported in the abstract with that reported in ClinicalTrials.gov when looking at the broad categories of treatment, prevention, diagnostic tests, and other (Table 1). However, there was less agreement when we compared more precise descriptions of the interventions. The interventions described in the abstract and the ClinicalTrials.gov record agreed exactly for 56% (88/154) of pairs, while 37% (57/154) of pairs agreed partly and 6% (9/154) of pairs did not agree (see Table 2 for examples of disagreements). In eight pairs, partial agreement involved small differences in the description of the intervention (e.g., 100 μl versus 110 μl of a drug), and in 49 pairs, partial agreement involved additional information about the intervention (e.g., dosage, time of administration, duration, etc.) in one source that was unavailable in the other. In 12 pairs, additional information was present only in the ClinicalTrials.gov record, and in 29 pairs additional information was present only in the abstract. For eight pairs, additional complementary information was present in both sources. Partial agreement also involved additional treatment arms: authors reported between one and four additional treatment arms in 11 ClinicalTrials.gov records and between one and six additional treatment arms in 25 abstracts.

**Table 2:** All interventions reported in ClinicalTrials.gov register and abstract that were classified as disagreements

	**Abstract**	**ClinicalTrials.gov**
**1**	1. lutein, zeaxanthin and long chain polyunsaturated fatty acids (LCPUFAs)	1. lutein, zeaxanthin, and 3 omega polyunsaturated fatty acid (PUFA)
	2. lutein and zeaxanthin	2. placebo for 3 omega (PUFA)
	3. LCPUFAs, docosahexanoic acid, and eicosapentanoic acid	3. placebo for lutein, zeaxanthin and 3 omega PUFA
		4. 4 mg triamcinolone
		5. 1 mg triamcinolone
		6. standard of care
**2**	1. 0.5D lens, head fixed	1. spectacle lenses
	2. 0.5D lens, head free	2. different spectacle lenses
	3. 1.0D lens, head fixed	
	4. 1.0D lens, head free	
	5. plano lens, head fixed	
	6. plano lens, head free	
**3**	1. folic acid, vitamin B6 and vitamin B12	1. aspirin ± vitamin E
	2. placebo	2. vitamin E ± vitamin C
		3. vitamin E ± vitamin C ± beta carotene ± folate
		4. placebo
**4**	1. photocoagulation using ETDRS grid	1. 810 nm diode laser photocoagulation
	2. photocoagulation with normal micro-pulsed technique	2. argon laser photocoagulation
	3. photocoagulation with high density micro-pulsed technique	
**5**	1. 0.3 mg ranibizumab monthly for 6 months	1. treatment with ranibizumab until resolution of macular edema or as macular edema occurs
	2. 0.5 mg ranibizumab monthly for 6 months	2. treatment with ranibizumab until resolution of edema and pigment epithelial detachment or as macular edema or pigment epithelial detachment occur
	3. 3 doses of ranibizumab, then treatment until macular fluid or pigment epithelial detachment absent	
**6**	1. laser photocoagulation	1. early treatment
	2. conventional treatment	2. standard of care
**7**	1. SofFlex IOL	1. Acrysof Natural lens
	2. SofPort IOL	2. AmoSensar Lens
**8**	1. plano lens plus weekend atropine	1. patching plus near activities at least one hour per day
	2. weekend atropine	2. atropine plus near activities at least one hour per day
	3. full spectacle correction plus weekend atropine	
	4. 2 hours daily patching	
**9**	1. contact lens care system with polyquaternium-1 and MAPD	1. silicone hydrogel contact lens
	2. contact lens care system with PHMB	2. 1XPMBH preserved MPS
		3. 1XPolyguad/Aldox MPS

### Masking or blinding

**Figure 3:**
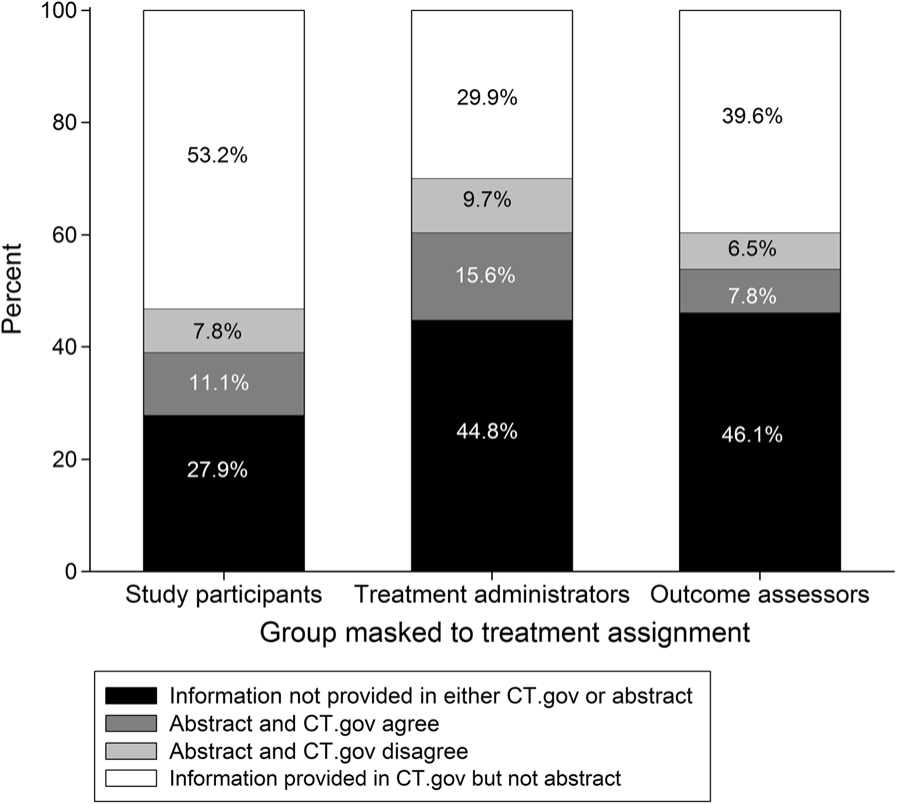
**Agreement between abstract and ClinicalTrials.gov register on masking by study role.** Bars show percent of abstract-ClinicalTrials.gov pairs that did not provide information on masking or blinding (black), agree (dark gray), or disagree (light gray), or where information was provided in the ClinicalTrials.gov record but not the conference abstract (white) on masking of study participants, persons administering the treatment, or persons measuring outcomes. Masking was categorized as present or not present. CT.gov = ClinicalTrials.gov.

We extracted information on masking separately for study participants, treatment administrators, and outcome assessors, finding that the ClinicalTrials.gov record frequently provided information on masking that was not available in the abstract (Figure 3).

### Sample size

The number of study participants or sample size was reported in both the abstract and ClinicalTrials.gov for the majority (136/154, 88%) of studies. There were eight studies in which a sample size was reported in the trials register, but not the abstract. We compared the reported sample sizes for pairs where the abstract author stated that the number of participants represented all randomized study participants and the status on the ClinicalTrials.gov record was either “completed” or “active, not recruiting” (see Table 3), and found good agreement.

**Table 3:** Comparison of number of randomized study participants reported in abstract with number reported in ClinicalTrials.gov

**Sample size comparisons for 66 abstract-ClinicalTrials.gov pairs**	
No. (%) pairs with exact match	20 (39)
No (%) pairs with percent difference^*^ ± 10%	18 (27)
No (%) pairs with percent difference^*^ > 10%	28 (42)
Range of percent differences	−106.6 to 88.7
Average (SD) percent difference	1.0 (32.9)

*Percent difference = ((No. participants reported in abstract – No. reported in ClinicalTrials.gov)/ No. participants reported in ClinicalTrials.gov) X 100.

Includes abstract-ClinicalTrials.gov pairs where number of participants in abstract included all randomized study participants and the status on the ClinicalTrials.gov record was either “completed” or “active, not recruiting.

### Outcomes

Authors reported 800 outcomes in 152 abstracts; no outcomes were reported in 2 abstracts. Thirty four percent (52/154) of abstracts and 82% (126/154) of ClinicalTrials.gov records explicitly described a primary outcome (Table 4). Of the 80 primary outcomes reported among the 40 abstract - ClinicalTrials.gov pairs, 14 (18%) were classified as being in complete agreement and 39 (49%) as partial agreement (see Table 5). Partial agreement typically involved a more explicit description of an outcome as shown in in Table 6. Of the remaining 27 primary outcomes reported in the abstract, 13 were reported elsewhere in the ClinicalTrials.gov record. There was poor agreement between non-primary outcomes reported in the abstract with those reported in ClinicalTrials.gov (see Table 5).

**Table 4:** **Agreement between conference abstract and ClinicalTrials.gov register on** r**eporting of one or more primary outcomes (n = 152 abstract-Clinical Trials.gov pairs)**

**Abstract**	**ClinicalTrials.gov**		
	**Reporting ≥ primary outcome**	**Not reporting a primary outcome**	**Total number of pairs**
	**No.**	**No.**	**No.**
**Reporting ≥ one primary outcome**	40	12	52
**Not reporting a primary outcome**	86	16	102
**Total number of pairs**	126	28	154

**Table 5:** Agreement between conference abstract and ClinicalTrials.gov register on outcomes (n = 40 abstract-ClinicalTrials.gov pairs reporting ≥ 1 primary outcome and 152 abstract-clinicaltrials.gov pairs reporting a non-primary outcome)

	**Primary Outcome**	**Non-primary outcome**
	**No. (%)**	**No. (%)**
**Total outcomes reported in abstract**	80 (100)	708 (100)
**Complete agreement**	14 (18)	57 (8)
**Partial agreement**	39 (49)	205 (29)
**Not reported in ClinicalTrials.gov**	27 (34)	446 (63)

**Table 6:** Examples of primary outcomes reported in ClinicalTrials.gov register with primary outcome reported in abstract classified as “partial agreement”

	**Abstract**	**ClinicalTrials.gov**	**Classified as**
**1**	Retinal thickness in the peak oedematous field on the retinal maps of the fast module scans of the Stratus OCT	Retinal thickness by fast retinal thickness mapping by optical coherence tomography at 0,2,4,8,10,15,20,30,60,90,120,and 180 min after last swallow of glycerol	Extra information in ClinicalTrials.gov
**2**	Visual acuity	Mean change in best corrected visual acuity as assessed by number letters read correctly on ETDRS eye chart at starting test distance of 4 meters from baseline to 1,3,6 months	Extra information in ClinicalTrials.gov
**3**	ETDRS best corrected visual acuity at 2, 6 and 12 weeks; OCT at 2 and 6 weeks; fluorescein angiography at 2 and 6 weeks	Best corrected visual acuity, OCT foveal thickness, fluorescein angiogram	Extra information in abstract
**4**	Change from baseline in major symptom complex score over period 1, consisting of runny nose, sniffles, itchy nose, nose blows, sneezes, and watery eyes	Mean change from baseline of major symptoms related to seasonal allergic rhinitis	Extra information in abstract
**5**	Retinal detachment rate; surgery-related complications; patient comfort; refractive change; visual acuity; OCT	Retinal detachment rate at 6 months	Extra complementary information in both sources

### Funders

One hundred forty-six unique funders were identified in either the abstract or the ClinicalTrials.gov record. At least one funder was reported in each ClinicalTrials.gov record, but only in 62% of abstracts (95/154). More than one funder was reported in 41 ClinicalTrials.gov records and in 19 abstracts. The same funder(s) were reported in both sources for 10 studies. We observed partial agreement across funders for 37 pairs, in which an additional or different funder was reported in either the abstract or in the ClinicalTrials.gov record.

### Contact information

The name of a contact person was included on 83% (128/154) of ClinicalTrials.gov records, but a phone number was found on only 25, and an email address on 26 records. No contact information was provided on any conference abstract except author name and affiliation.

## Discussion

A substantial amount of additional information on study design was available on the ClinicalTrials.gov record that was not presented in a corresponding conference abstract. Information on multi-center status, eligibility criteria with respect to sex, who is masked, and primary outcome is present to varying degrees in the ClinicalTrials.gov register record. In addition, the name of a contact was included in 83% of ClinicalTrials.gov records, so that if desired information about the trial was not available, or conflicted with what was reported in the abstract, a systematic reviewer could contact the study author directly, although contact information was provided in a minority of ClinicalTrials.gov records.

Thus, if a trial registration number is available for a study to be included in a systematic review, a systematic reviewer may be able to find additional information about study design in a trials register record. However, there are caveats to these findings. First, information about trial registration may not be required nor provided by conference organizers generally. Second, we encountered numerous disagreements between the information provided in the conference abstract with information contained within the ClinicalTrials.gov record on a number of items. ClinicalTrials.gov was originally intended to provide a source of information about the existence of a trial for patients and clinicians, to diminish redundant research effort, and to alert researchers to the possibility of publication bias. Currently it provides limited protocol information, although it would be a natural repository for full protocols. A published protocol or design and methods paper would provide more information, but until public availability of all trial protocols is achieved, trials registers serve as surrogates for study protocols.

The findings from our study relate specifically to ARVO abstracts and ClinicalTrials.gov and may not be applicable to other conference abstracts or trials registers. Furthermore, our requirement that a valid ClinicalTrials.gov registration number be reported on the abstract submission form could mean that studies were actually registered but the number not recorded or incorrectly recorded.

Previous studies have identified discrepancies between trials register records and associated full length publication on study design characteristics, especially related to the description of primary and secondary outcomes [11-16]. In our study, we found discrepancies related to the detailed descriptions of study interventions and outcomes reported in the conference abstract compared with those reported in the ClinicalTrials.gov record. Frequently there was more information available in the abstract than was in the ClinicalTrials.gov record (e.g., dose or duration of treatment). Most disturbing was the appearance of additional treatment arms in the abstract that were not included in the register, suggesting that investigators are not regularly updating the trial register record as required or that abstracts represent preliminary findings, before an arm was dropped.

The discrepancies in outcomes may be an indication of error, selective outcome reporting, or simply due to investigators not updating the ClinicalTrials.gov record in a timely manner. We obtained register records that were available shortly following presentation of abstract results at ARVO, so any additional outcomes or changes in the study design should have been incorporated into the trial register as an amendment to the protocol items. In addition, we compared outcomes in only one direction, i.e. from abstract to Clinicaltrials.gov record. We did not do the reverse analysis (i.e. comparing all outcomes reported in ClinicalTrials.gov with those reported in the abstract) because we would not expect an abstract to include all outcomes reported in ClinicalTrials.gov. In so doing, we did find that sometimes the outcome reported in the abstract as the “primary” outcomes was a secondary outcome in the ClinicalTrials.gov record. In the future it will be of interest to compare outcomes reported as results with the specified outcomes in the ClinicalTrials.gov record. Results reporting in ClinicalTrials.gov opened in September 2008. When we retrieved the records in May and June 2009, none of the records included any study results when downloaded.

Other studies have also found discrepancies between the description of an RCT as reported in an abstract with what was reported in a full length publication [17-21]. It would be of interest to make a three-way comparison of a conference abstract, trial register and results record, and full length publication to determine congruence between these sources of information. It would also be useful for authors to include conference abstracts in the ClinicalTrials.gov record as a publication, in addition to full length publications.

Whether the study results reported in an abstract should be included in a systematic review or used to make clinical decisions is unclear. That results from abstracts are being included in systematic reviews has been reported in a sample of meta-analyses indexed in Medline [7], for health technology assessments [5], and for drug formulary decision-making [22]. Including abstracts in systematic reviews is recommended by IOM [1], the Cochrane Collaboration [23], and the Agency for Health Care Research and Quality [24], although caution is urged due to the preliminary nature of abstract results [25,26]. Results from a Cochrane systematic review found that there was an overall smaller treatment effect if systematic reviewers included abstract results in the review compared to the treatment effect size without abstracts included, although this finding was not statistically significant [3]. Additional investigators report similar results, showing small reductions in the size of the treatment effect when abstract results are included in a systematic review [27,28].

Overall, given our findings, we would encourage systematic reviewers to take advantage of the information provided in the ClinicalTrials.gov record to supplement the information provided in an abstract. We did not record the amount of time it took to extract information for each record. Because the trials register number was available, matching the abstract to the registry report was straightforward. Extracting all the information we did for our study was somewhat time consuming, and we found that it was more efficient to obtain the information from the tabular view in ClinicalTrials.gov rather than the full text view. Most likely, it would take much less time for a systematic reviewer looking for specific information. We would caution systematic reviewers to exercise care in using data from a trials register record, however, as with any unpublished study result. In many cases, systematic reviewers may not be able to distinguish between conflicting reports and still find it necessary to contact the study investigators. For this situation, the ClinicalTrials.gov record frequently provides contact information that is almost always missing from a conference abstract.

## Conclusions

Systematic reviewers may find additional information about an RCT in the ClinicalTrials.gov record to supplement the information provided in a conference abstract. However, it may still be necessary to contact study investigators for information not included in either the abstract or ClinicalTrials.gov record, or that is in conflict between the two sources.

## Abbreviations

ARVO: Association for Research in Vision and Ophthalmology; CONSORT: Consolidated Standards of Reporting Trials; IOM: Institute of Medicine; RCT: Randomized controlled trial.

## Competing interests

The authors declare that they have no competing interests.

## Authors’ contributions

RWS conceived of the study, participated in the study design and data extraction, entered the data in the Access database, participated in the analysis and interpretation of results, and drafted the manuscript. LH participated in the study design, data extraction, analysis and interpretation of results. AE participated in the study design, data extraction, and interpretation of results. JT participated in the study design and data extraction. KD participated in data extraction and interpretation of results. All authors read, edited, and approved the final manuscript.

## Pre-publication history

The pre-publication history for this paper can be accessed here:

http://www.biomedcentral.com/1471-2288/13/79/prepub

## References

[B1] IOM (Institute of Medicine) Knowing What Works in Health Care: a Roadmap for the Nation. Committee on Reviewing Evidence to Identify Highly Effective Clinical Services 2011 Washington DC: The National Academic Press

[B2] Scherer RW Langenberg P Von Elm E Full publication of results initially presented in abstracts Cochrane Database Syst Rev 2007 2 Art. No.: MR000005 10.1002/14651858.MR000005.pub3 17443628

[B3] Hopewell S McDonald S Clarke MJ Egger M Grey literature in meta-analyses of randomized trials of health care interventions Cochrane Database Syst Rev 2007 2 Art. No.:MR000010 10.1002/14651858.MR000010.pub3 PMC8973936 17443631

[B4] Hopewell S Eisinga A Clarke M Better reporting of randomized trials in biomedical journal and conference abstracts J Information Science 2008 34 162 173

[B5] Dundar Y Dodd S Williamson P Walley T Dickson R Searching for and use of conference abstracts in health technology assessments: Policy and practice Int J Technol Assess Health Care 2006 22 283 287 16984054 10.1017/s0266462306051154

[B6] Egger M Juni P Bartlett C Holensteing F Sterne J How important are comprehensive literature searches and the assessment of trial quality in systematic reviews? Empirical study Health Technol Assess 2003 7 Issue 1 1 76 12583822

[B7] Cook DJ Guyatt GH Ryan G Clifton J Buckingham L Willan A McIlroy Oxman AD Should unpublished data be included in meta-analyses? JAMA 1993 269 2749 2753 10.1001/jama.1993.03500210049030 8492400

[B8] Association for Research in Vision and Ophthalmology Statement on Registering Clinical Trials http://www.arvo.org/About_ARVO/Policies/Statement_on_Registering_Clinical_Trials

[B9] Scherer RW Sieving P Dickersin K Can we depend on investigators to identify and register randomized controlled trials? PLoS One 2012 7 e44183 10.1371/journal.pone.0044183 22984474 PMC3439467

[B10] Training Manual for Handsearchers, US Cochrane Center [http://us.cochrane.org/sites/us.cochrane.org/files/uploads/pdf/Handsearcher%20Training%20Manual.pdf]

[B11] Dwan K Altman DG Cresswell L Blundell M Gamble CL Williamson PR Comparison of protocols and registry entries to published reports for randomised controlled trials Cochrane Database Syst Rev 2011 Issue 1 Art. No.: MR000031 10.1002/14651858.MR000031.pub2 PMC7390503 21249714

[B12] Ghandi R Jan M Smith HN Mahomed NN Bhandari M Comparison of published orthopaedic trauma trials following registration in Clinicaltrials.gov BMC Musculoskelet Disord 2011 12 278 At. http://biomedcentral.com/1471-2474/12/278 10.1186/1471-2474-12-278 22151841 PMC3266218

[B13] You B Gan HK Pnd G Chen EX Consistency in the analysis and reporting of primary end points in oncology randomized controlled trials form registration to publication: a systematic review J Clin Oncol 2012 30 210 216 10.1200/JCO.2011.37.0890 22162583

[B14] Mathieu S Boutron I Moher D Altman DG Ravaud P Comparison of registered and published primary outcomes in randomized controlled trials JAMA 2009 302 977 984 10.1001/jama.2009.1242 19724045

[B15] Millette K Roseman M Thombs BD Transparency of outcome reporting and trial registration of randomized controlled trials in top psychosomatic and behavioral health journals: a systematic review J Psychosomatic Research 2011 70 205 217 10.1016/j.jpsychores.2010.09.015 21334491

[B16] Bourgeois F Murthy S Mandl KD Outcome reporting among drug trials registered in ClinicalTrials.gov Ann Intern Med 2010 153 158 166 10.7326/0003-4819-153-3-201008030-00006 20679560 PMC3374868

[B17] Toma M McAlister FA Bialy L Adams D Vendermeer B Armstrong PW Transition from meeting abstract to full-length journal article for randomized controlled trials JAMA 2006 295 1281 1287 10.1001/jama.295.11.1281 16537738

[B18] Dundar Y Dodd S Williamson P Dickson R Walley T Case study of the comparison of data from conference abstracts and full-text articles in health technology assessment of rapidly evolving technologies: Does it make a difference? Int J Tech Assess Health Care 2006 22 288 294 10.1017/s0266462306051166 16984055

[B19] Prasad S Lee DJ Yuan JC-C Barao VAR Shyamsundeer N Sukotjo C Discrepancies between abstracts presented at International Association for Dental Research annual sessions from 2004 to 2005 and full-text publications International J Dentistry 2012 859561 10.1155/2012/859561 PMC3296196 22505912

[B20] Tam VC Hotte SJ Consistency of phase III clinical trial abstracts presented at an annual meeting of the American Society of Clinical Oncology compared with their subsequent full-text publications J Clin Oncol 2008 26 2205 2211 10.1200/JCO.2007.14.6795 18445846

[B21] Snedeker KG Campbell M Totton SC Guthrie A Sargeant JM Comparison of outcomes and other variables between conference abstracts and subsequent peer-reviewed papers involving pre-harvest or abbatoir-level interventions against foodborne pathogens Prev Vet Med 2010 9 67 76 20739075 10.1016/j.prevetmed.2010.07.012

[B22] Weizman AV Griesman J Bell CM The use of research abstracts in formulary decision making by the Joint Oncology Drug Review of Canada Appl Health Econ Policy 2010 8 387 391 10.2165/11530510-000000000-00000 21043540

[B23] Cochrane Handbook of Systematic Reviews of Interventions [http://handbook.cochrane.org/]

[B24] Relevo R Balshem H Finding evidence for comparing medical interventions: AHRQ and the Effective Health Care Program J Clin Epidem 2011 64 168 1177 10.1016/j.jclinepi.2010.11.022 21684115

[B25] Falagas ME Rosmarakis ES Clinical decision-making based on findings presented in conference abstracts: is it safe for our patients? Eur Heart J 2006 27 2038 2039 10.1093/eurheartj/ehl175 16864607

[B26] Wong SS-W Fraser C Lurenco T Barnett D Avenell A Glazener C Cuthberston B N’Dow J The fate of conference abstracts: systematic review and meta-analysis of surgical treatments for men with benign prostatic enlargement World J Urol 2010 28 63 69 10.1007/s00345-009-0500-3 20049457

[B27] Hoehner C Soares J Parra DC Ribeiro IC Pratt M Bracco M Hallal PC Bownson RC Physical activity interventions in Latin America: What value might be added by including conference abstracts in a literature review? J Physical Activity and Health 2010 7 Suppl 2 S265 S278 10.1123/jpah.7.s2.s265 20702915

[B28] Hopewell S Clarke M Moher D Wager E Middleton P Altman DG Schulz KF CONSORT Group CONSORT for reporting randomized controlled trials in journal and conference abstracts: Explanation and elaboration PLoS Med 2008 5 e20 10.1371/journal.pmed.0050020 18215107 PMC2211558

